# Recent Advances in Membrane Shaping for Plant Autophagosome Biogenesis

**DOI:** 10.3389/fpls.2020.00565

**Published:** 2020-05-28

**Authors:** Cheuk-Ling Wun, Yingfei Quan, Xiaohong Zhuang

**Affiliations:** School of Life Sciences, Centre for Cell & Developmental Biology and State Key Laboratory of Agrobiotechnology, The Chinese University of Hong Kong, Hong Kong, China

**Keywords:** autophagosome, membrane tethering, lipid transfer, vesicle fusion, membrane shaping

## Abstract

Autophagy is an intracellular degradation process, which is highly conserved in eukaryotes. During this process, unwanted cytosolic constituents are sequestered and delivered into the vacuole/lysosome by a double-membrane organelle known as an autophagosome. The autophagosome initiates from a membrane sac named the phagophore, and after phagophore expansion and closure, the outer membrane fuses with the vacuole/lysosome to release the autophagic body into the vacuole. Membrane sources derived from the endomembrane system (e.g., Endoplasmic Reticulum, Golgi and endosome) have been implicated to contribute to autophagosome in different steps (initiation, expansion or maturation). Therefore, coordination between the autophagy-related (ATG) proteins and membrane tethers from the endomembrane system is required during autophagosome biogenesis. In this review, we will update recent findings with a focus on comparing the selected core ATG complexes and the endomembrane tethering machineries for shaping the autophagosome membrane in yeast, mammal, and plant systems.

## Introduction

Macroautophagy (hereafter referred to as autophagy) is an evolutionarily conserved degradative process required for maintaining cellular homeostasis. In this pathway, detrimental or unwanted cellular materials are engulfed by a *de novo* formed double-membrane autophagosome and delivered into the vacuole (in yeast and plants) or lysosome (in animals) for degradation/recycling (Mizushima et al., 2011; Liu and Bassham, 2012). The autophagosome biogenesis process can be divided into four major stages: initiation of a cup-shaped membrane sac (phagophore/isolation membrane), expansion of the phagophore, autophagosome closure, and autophagosome-vacuole/lysosome fusion (Soto-Burgos et al., 2018; Zhuang et al., 2018). To form a mature autophagosome, a number of autophagy-related (ATG) proteins are employed to orchestrate the membrane shaping of the autophagosome in the different steps of autophagy (Soto-Burgos et al., 2018; Zhuang et al., 2018). In addition, membrane tethers from the endomembrane system have also been implicated to participate in membrane remodeling processes during autophagosome biogenesis. Exciting findings have shed new light on the non-ATG regulators in shaping the autophagosome membrane in plants, such as cytoskeleton, phospholipids, and membrane tethering machineries (e.g., the exocyst complex), which have been reviewed in several recent excellent publications (Tzfadia and Galili, 2013; Pecenkova et al., 2017; Wang et al., 2017; Soto-Burgos et al., 2018; Chung, 2019) and therefore will not be covered here. In this mini review, we will discuss the recent advances of the core ATG machineries as well as the related endomembrane tethering machineries in shaping the autophagosome membrane (Table 1).

**TABLE 1:** Core regulators in autophagosome biogenesis in yeast, mammals, and plants*.

Process	Complex/protein	Pivotal subunits/proteins			Putative roles in Sc and Hs	Putative roles in At
		Sc	Hs	At		
Phagophore	Atg1 complex	Atg1	ULK1/2	ATG1 (a, b, c, t)	Ser/Thr kinase	Ser/Thr kinase
initiation		Atg17-Atg31-Atg29	FIP200	ATG11	Membrane scaffold	/
		Atg13	ATG13	ATG13a/b	ATG9 vesicle recruitment	/
	TRAPP complex	Trs85	TRAPPC8	TRS85	GEF effector for Ypt1/RAB1 activation	/
		Trs33	–	TRS33		/
	GTPase	Ypt1	RAB1A/B	RAB1/RABD	Atg1 and ATG9A vesicle recruitment	/
Phagophore	Atg9 complex	Atg2	ATG2A/B	ATG2	Lipid transfer, ATG9 vesicle recruitment	/
expansion		Atg18	WIPI1/2/3/4	ATG18a-h	PI3P effector	PI3P effector
		Atg9	ATG9A/B	ATG9	Lipid source	Lipid source
	PI3K complex	Atg14-Atg6-Vps34-Vps15	ATG14L-Beclin1-VPS34-VPS15	ATG14a/b-ATG6-VPS34-VPS15	PI3P generation	PI3P generation
AP closure	ATG8-PE	Atg8	LC3A/B/C, GABARAP, GABARAPL1/L2	ATG8a-i	Cargo recognition; Membrane tethering and hemifusion	Cargo recognition
	ESCRT complex	/	ESCRT-I (VPS37A)	ESCRT-I (FREE1)	Assembly of ESCRT complex	Assembly of ESCRT complex
		ESCRT III (Snf7, Vps4)	ESCRT-III (CHMP2A)	ESCRT-III (CHMP1)	Membrane scission	Membrane scission
	GTPase	Rab5	RAB5A/B/C	RAB5 (ARA6/7, RHA1/RABF2a)	Recruitment of ESCRT to PAS	/
AP-vacuole/	SNARE complex	Vti1-Vam3-Vam7-YKT6	STX17-SNAP29-VAMP7/8	VTI11/12/13	Membrane tethering, recruitment of HOPS complex	Membrane tethering, recruitment of HOPS complex
lysosome fusion		/	STX7-SNAP29-YKT6	/	Membrane tethering, recruitment of HOPS complex	/
	HOPS complex	Vps39, Vps41	VPS39, VPS41	VPS39, VPS41	Vesicles fusion	/
	Mon1-Ccz1 complex	Mon1-Ccz1	Mon1-Ccz1	MON1-CCZ1	GEF complex for Rab7 GTPase activation	GEF complex for Rab7 GTPase activation
	GTPase	Ypt7	RAB7a/b	RAB7 (RABG3a-f)	HOPS complex recruitment	HOPS complex recruitment

*Not all the regulators are listed in the table. For orthologs with no evidence supporting their roles in specific steps of autophagy or not identified (/). At, *Arabidopsis thaliana*; Hs, *Homo sapiens*; Sc, *Saccharomyces cerevisiae*; AP, autophagosome.

## Phagophore Initiation

**FIGURE 1:**
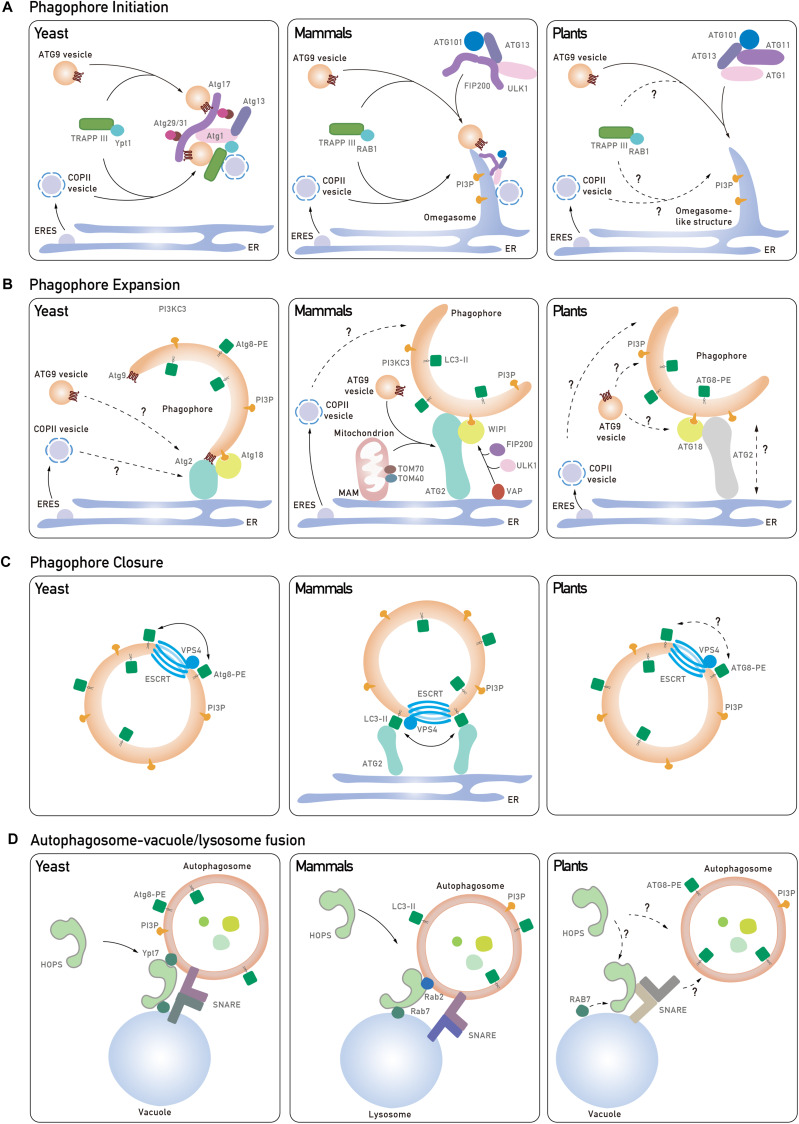
Core tethering machineries in autophagosome biogenesis in yeast, mammals, and plants. **(A)** The phagophore initiation is mediated by the recruitment of Atg1 complex to the pre-autophagosomal structure (PAS) in yeast. Atg9 vesicles can interact with Atg1 complex and provide a membrane source by nucleation to form a cup-shape phagophore. In mammals, trafficking of ATG9 to PAS is mediated by the TRAPPIII complex, and the S-shaped ULK1 complex is recruited to the ATG9-positive ER membrane for phagophore initiation. Another membrane source, COPII vesicles, are involved in the initiation process in both yeast and mammals. In plants, the ER is one of the assembly sites for phagophore initiation, which requires the ATG1 complex and ATG9 vesicles, but the roles of the TRAPPIII complex and COPII vesicles remain unknown (dashed and question mark). **(B)** In yeast, Atg9 directs the Atg2-Atg18 complex onto the phagophore, which mediates the formation of IM-ER contacts for phagophore expansion. In mammals, WIPI2 interacts with ULK1/FIP200-VAPs complex assembled on the ER membrane to mediate the IM contact with the ER. ATG2-WIPI4 complex interacts with mitochondrial proteins to establish the IM-MAM contact and to recruit ATG9 vesicles to MAM. In plants, ATG18 transiently associates with ATG9 but the function of ATG18-ATG2 in phagophore extension remains unknown (dashed and question mark). In addition, COPII vesicles have been implicated to contribute to phagophore expansion, although their role in plants is unclear (dashed and question mark). **(C)** Atg8 and ESCRT complex are potential candidates in regulating autophagosome closure. Atg8-PE is likely to promote hemifusion by tethering lipid membrane or another Atg8-PE for the closure process in both yeast and mammals. But it is also suggested that specific LC3 isoforms interact with ATG2 which are anchored to the ER to close the autophagosome in mammals. ESCRT subunits might also participate in the final fission process to seal the autophagosome. **(D)** In yeast and mammals, distinct pairs of SNAREs are recruited to the autophagosomal membrane and the vacuole/lysosome, respectively. Upon the activation of GTPase, the HOPS complex is recruited to assist in SNARE complex assembly and tethering events for autophagosome-vacuole/lysosome fusion. In *Arabidopsis*, how Rab GTPase, SNARE complex and HOPS complex are coordinated to regulate autophagosome-vacuole fusion remains elusive (dashed and question mark).

In yeast, autophagy is initiated as a pre-autophagosomal structure (PAS) by the hierarchical recruitment of a number of Atg proteins, in particular the Atg1 complex and Atg9 (Yamamoto et al., 2016). The PAS in yeast is often detected in close proximity to both the ER and the vacuole, and a vesicle delivery model has been proposed, contributed by Atg9 vesicles and COPII vesicles (Figure 1A, left; Otomo et al., 2018; Hollenstein et al., 2019). Key players like Ypt1 and the transport protein particle (TRAPP) GEF (guanine-nucleotide exchange factors) complex are both reported to participate in this process (Lipatova et al., 2016). In yeast, 4 types of TRAPP complex (TRAPPI-IV) have been identified, and most homologs of TRAPP subunits are also found in mammal and plants (Lipatova et al., 2016). Upon autophagic induction, the TRAPPIII complex consisting Trs85 is recruited to the PAS for the activation of Ypt1 (Lynch-Day et al., 2010). Of note, it is shown that Trs33, which was originally considered to be a subunit exists in all TRAPP complexes, may assemble into a distinct TRAPP complex in the absence of Trs85 for Ypt1-mediated autophagy as well (Lipatova et al., 2016). Subsequently, Ypt1 triggers the assembly of Atg1-Atg13-Atg17-Atg31-Atg29 complex on the PAS for the clustering of Atg9 vesicles and COPII vesicles to produce a membrane sac (Figure 1A, left). Structural studies have revealed that the Atg17-Atg31-Atg29 subcomplex forms an S-shaped scaffold to bridge two Atg9 vesicles together, while such a crescent-shape structure perfectly matches the size of Atg9 vesicles (∼20–30 nm) (Ragusa et al., 2012). Additionally, COPII subunits also bind to Ypt1 and Atg9, as well as the Atg17-Atg31-Atg29 tethering complex for phagophore initiation (Wang et al., 2014).

Unlike yeasts which contain a single PAS, multiple PAS have been detected in mammalian cells. The current model for phagophore nucleation involves an intimate membrane association among an omega-shape structure called the omegasome, which emerges from the ER, as well as ATG9 vesicles and COPII vesicles (Figure 1A, middle; Axe et al., 2008). It appears that ATG9 vesicles are more likely to supply proteins/lipids, instead of being directly incorporated into the phagophore membrane. This is supported by a recent study showing that ATG9 vesicles deliver the PI4-kinase, PI4KIIIβ, to the autophagosome initiation site for the recruitment of the ULK1/2 complex (counterpart of yeast ATG1 complex) (Judith et al., 2019). In addition, the TRAPPIII complex in mammal is required for the relocalization of ATG9 vesicles from peripheral recycling endosomes to the early Golgi during autophagy (Lamb et al., 2016). Using high-resolution imaging analysis, it has been observed that ULK and FIP200 are recruited onto the ER membrane prior to their association with the ATG9 vesicles (Karanasios et al., 2016). After that, the ULK1 complex translocates to the ATG9A-positive autophagosome precursors in a PI3P-dependent manner, while ATG9 is phosphorylated by ULK1, thus further promoting ATG9 trafficking under starvation conditions (Zhou et al., 2017). Interestingly, recently it is showed that FIP200 dimerizes as a C-shaped hub for the assembly of the ULK1 complex, but whether the C-shape would fit the ATG9 vesicles to promote phagophore nucleation remains an open question (Shi et al., 2019).

In plant cells, omegasome-like structures have also been observed during autophagy (Zhuang et al., 2013) and conserved ATG1 complex subunits have been identified in the plant genome (Huang et al., 2019; Figure 1A, right). It has been reported that mutation of all ATG1 isoforms significantly compromised autophagosome formation (Huang et al., 2019). Conversely, depletion of plant ATG9 results in abnormal ATG8-positive tubules connecting to the PI3P-enriched ER subdomain, and similar defects are also observed in *atg11* mutant, indicating that they both act downstream of ATG1 to organize phagophore assembly (Zhuang et al., 2017; Huang et al., 2019). Interestingly, structural analysis showed that ATG9 may form a trimer, suggesting that ATG9 may recruit additional ATG9-containing vesicles via self-tethering during phagophore nucleation (Lai et al., 2019). However, to get a better understanding of how the ATG1 complex and ATG9 vesicles are assembled at the initiation site, future investigations are needed to examine their hierarchical order (Figure 1A, right).

## Phagophore Expansion

Later the phagophore will elongate to expand its membrane size for cargo sequestration, and this also requires a subset of membrane remodeling proteins to transport lipids/proteins from the membrane donors. Membrane donors including the ER, ATG9 vesicles, COPII vesicles, as well as other endosomes have also been implicated as contributing to phagophore elongation (Gomez-Sanchez et al., 2018; Osawa et al., 2019; Shima et al., 2019; Figure 1B, left). During phagophore expansion, the PI3K complex mediates the production of PI3P, which further recruits PI3P effectors (e.g., ATG18), as well as the ATG12-ATG5-ATG16 conjugate (Simonsen and Tooze, 2009; Romanov et al., 2012). Afterward, cytosolic ATG8 is conjugated to phosphatidylethanolamine (PE) to form ATG8-PE (LC3-II in mammal) that decorates the phagophore, which may contribute to cargo recognition and phagophore closure (discuss later) (Fujita et al., 2008). In yeast, Atg8 may function in autophagosome expansion, as mutation of *atg8* leads to reduced-sized or even no autophagosome production (Xie et al., 2008).

Recent studies have demonstrated that the isolation membrane (IM) expands via lipid transfer from the ER at the IM-ER contact (Gomez-Sanchez et al., 2018). In yeast, Atg2-Atg18 interacts with Atg9 vesicles to mediate Atg9 vesicle recycling, while ATG9 directs Atg2 to the IM-ER contact sites (Figure 1B, left; Gomez-Sanchez et al., 2018). Loss of Atg2-Atg9 interaction compromises the formation of the IM-ER contact site (Gomez-Sanchez et al., 2018). Interestingly, Atg2 also recognizes PI3P, and Atg18 alone cannot bind to PI3P on IM unless Atg2 is present (Gomez-Sanchez et al., 2018; Kotani et al., 2018). Mutation of the Atg2 lipid-binding site leads to shorter IM (Osawa et al., 2019). Moreover, fluorescence-based liposome binding assays have showed that Atg2 has a stronger membrane tethering ability to small liposomes (with high curvature) that are consistent with the size of Atg9 vesicles, suggesting that Atg9 vesicles might be the membrane donor in phagophore expansion (Osawa et al., 2019). Similar to the lipid transport protein vacuolar protein sorting 13 (Vps13), Atg2 also contains a hydrophobic cavity in the conserved N-terminal region, which allows Atg2 to solubilize lipid for lipid transfer (Kumar N. et al., 2018; Osawa et al., 2019).

The ability of lipid transfer is conserved in mammalian ATG2. Structural analysis has revealed that ATG2 is a rod-like protein that forms a complex with WIPI1 (WD repeat domain phosphoinositides-interacting protein 1), WIPI2 and WIPI4 (mammalian homologs of Atg18) to bridge IM-ER contact (Chowdhury et al., 2018; Maeda et al., 2019). Unidirectional lipid transfer from the ER to the PI3P-enriched IM is facilitated by ATG2 with the assistance of WIPI proteins (Figure 1B, middle; Chowdhury et al., 2018; Maeda et al., 2019). In addition, ER receptor vesicle-associated membrane protein-associated proteins (VAPs) have also been reported to mediate the IM-ER contact formation via interaction with FIP200 and ULK1 (Zhao et al., 2018). Then, WIPI2 tethers the ER with the IM via binding to PI3P on IM and the ULK1/FIP200-VAPs on the ER (Figure 1B, middle). VAPs probably stabilize the complex for membrane contacts and loss of VAPs results in impairment of phagophore growth (Zhao et al., 2018). Of note, a recent study also reported accumulation of ATG2 at mitochondria-associated membrane (MAM) (Tang et al., 2019). It has been suggested that after having been directed to MAM by TOM40 on mitochondria, ATG2 will recruit ATG9 vesicles to mediate lipid transfer from ATG9 vesicles to the IM (Figure 1B, middle; Tang et al., 2019). This is supported by the observation that loss of ATG2-ATG9 interaction resulted in a failure of ATG9 vesicle delivery and defects in phagophore expansion (Tang et al., 2019).

Arabidopsis *atg2*, *atg9*, and *atg18* mutants all show defects in autophagosome formation and display an early senescence phenotype, implying that a conserved plant ATG2-ATG18-ATG9 complex might function in autophagosome expansion as well (Figure 1B, right) (Hanaoka et al., 2002; Zhuang et al., 2017; Kang et al., 2018). Particularly, the levels of both the unlipidated and lipidated ATG8 are significantly increased in all *atg2*, *atg9*, and *atg18* mutants, suggesting a failure in autophagosome formation (Kang et al., 2018). It is worth noting that in the *atg2* mutant, numerous tiny autophagic structures are detected in the cytosol and their delivery into the vacuole are blocked (Kang et al., 2018). Intriguingly, extending tubules positive with both ATG8 and ATG18, which are sensitive to PI3P inhibitor, are accumulated in *atg9* mutant (Zhuang et al., 2017). One possible scenario is that ATG9 vesicles may regulate the retrieval of ATG18 and ATG2 from the PAS to prevent excessive expansion of the phagophore.

## Autophagosome Closure

The closure of the phagophore into an autophagosome is a process involving outer membrane fusion along a “rim of a cup,” while an inner autophagic vesicle is separated from the outer membrane, which is topologically identical to the membrane fission process (Zhou et al., 2019).

In yeast, Atg8-PE itself has been shown to facilitate membrane tethering and hemifusion (Figure 1C, left) (Nakatogawa et al., 2007). In mammalian cells, a similar function for the LC3/GABARAP protein family (the homolog of Atg8) in autophagosome closure has been reported. Knockdown of all six LC3/GABARAP isoforms results in the accumulation of open IMs (Nguyen et al., 2016). Moreover, inhibiting LC3 lipidation also induced the accumulation of open autophagic structures (Fujita et al., 2008). Structural analysis showed that the unique N-terminal region of LC3/GABARAP can bind to both lipid and protein (e.g., another LC3/GABARAP) (Wu et al., 2015), suggesting a multimerization ability of the LC3/GABARAP family for hemifusion. Interestingly, another study revealed a distinct role of ATG2-GABARAP interaction in autophagosome closure (Bozic et al., 2019). Both ATG2A and ATG2B contain LC3 interacting region (LIR) motifs for interaction with LC3/GABARAP. In *atg2a/atg2b* double knock out cells, or even expressing mutated ATG2A lacking the interaction region to GABARAP, the number of unsealed autophagosomes is significantly increased. However, such defects cannot be complemented in *atg2a/atg2b* double knock out cells expressing ATG2 which abolishes WIPI4 interaction. Therefore, the ATG2-GABARAP interaction might represent a novel mechanism in autophagosome closure, which is independent of the association of ATG2-WIPI in phagophore expansion. In plants, the great expansion of ATG8 family, which comprises 9 isoforms, hinders the characterization of the specific roles of different ATG8 isoforms in autophagosome formation (Liu and Bassham, 2012). However, future efforts are needed to utilize different tools like *in vitro* assays and structural analysis to obtain more detailed information on the ATG8 family in plants.

The ESCRT machinery has been well characterized to function in reverse topology scission of membrane, particularly for the formation of the internal vesicles in the multivesicular body (MVB) (Gao et al., 2017). In regard to the similar topology for the invagination of the internal vesicles from the outer membrane, the ESCRT complex would be a good membrane scission candidate for sealing the autophagosome membrane (Figure 1C). Indeed, in most ESCRT mutants, abnormal autophagosome structures are often detected in the cytosol (Takahashi et al., 2018, 2019; Zhou et al., 2019). Recently, using a novel method to distinguish the phagophore and nascent autophagosome by labeling LC3 with permeable and impermeable dyes, it was shown that an ESCRT-III component CHMP2A and an AAA-ATPase vacuolar protein sorting-associated 4 (VPS4) translocate to the phagophore during autophagy (Takahashi et al., 2018). Furthermore, more unsealed phagophore structures and protease-unprotected GFP-LC3-II are detected in *chmp2a* and *vps4* cells. Recently, another ESCRT-I subunit VPS37A, has been identified to recruit the ESCRT machinery onto the phagophore to mediate its closure (Takahashi et al., 2019). Another strong piece of evidence from yeast study is that Atg17 binds to Snf7 for its recruitment to the phagophore in a Rab5-dependent manner (Zhou et al., 2019). Depletion of Snf7, Vps4 and Rab5 GTPase Vps21 all result in an accumulation of immature autophagosomes. Particularly, with an artificially forced Atg17–Snf7 interaction, no defects are displayed in a *vps21* mutant, suggesting that Rab5 GTPase catalyzes the Atg17–Snf7 interaction for autophagosome closure.

In plants, the essential roles of ESCRT machinery in plant MVB-mediated pathways have been well documented and abnormal autophagosomes are accumulated in several ESCRT-related mutants as well (Gao et al., 2017). For instance, in the *chmp1* mutant, delayed autophagosome closure as well as abnormal pattern in plastid division is observed, indicating that CHMP1 may function in closure of autophagosome for sequestering plastid cargo (Spitzer et al., 2015). Autophagosome maturation and delivery into the vacuole are also severely suppressed when another plant unique ESCRT subunit, FREE1 is mutated. Further evidence demonstrates a direct link between the ESCRT machinery and autophagic machinery via a FREE1-SH3P2 interaction (Gao et al., 2015), whereby SH3P2 has been shown to interact with ATG8 and to translocate onto the phagophore upon autophagic induction (Zhuang et al., 2013). However, the regulatory mechanism of plant ESCRT in autophagosome closure awaits further investigations (Figure 1C, right).

## Autophagosome-Vacuole/Lysosome Fusion

In the endomembrane system, membrane tethering machineries, including the soluble N-ethylmaleimide-sensitive factor attachment receptor (SNARE) complex and homotypic fusion and vacuole protein sorting (HOPS) complex, have been well characterized to mediate the fusion between the vacuole/lysosome and other vesicles (Itakura et al., 2012; Jiang et al., 2014; Bas et al., 2018; Matsui et al., 2018).

The SNARE complex can change its conformation to shorten the distance between two compartments and facilitate membrane insertion into the target membrane (Jahn and Scheller, 2006). Two types of SNAREs, Q-SNAREs and R-SNARE, which are localized on the membrane acceptor and the membrane donor, respectively, assemble into a four-helix SNARE complex and recruit the HOPS complex (Jahn and Scheller, 2006). In yeast, the Q-SNARE on the vacuole, comprised of Vti1, Vam3, Vam7, cooperates with the R-SNARE on autophagosome, Ykt6, to mediate the autophagosome/vacuole fusion (Bas et al., 2018). The HOPS complex, which is highly conserved in yeast, mammals, and plants, is characterized as an elongated seahorse-like tether (Brocker et al., 2012). To activate the HOPS complex, Rab7-like GTPase Ypt7 is recruited onto the autophagosome membrane via the Mon1-CcZ1 (monensin sensitivity protein 1 -caffeine, calcium, and zinc 1) GEF complex, which is conserved in yeast, mammals and plants (Cui et al., 2014; Gao et al., 2018). Importantly, Ccz1 consists of an LC3-interacting region (LIR) motif which interacts with Atg8 on the autophagosome, activating Ypt7 for its recruitment via binding to PI3P on the autophagosome (Gao et al., 2018). However, mutation of the PI3K subunits Atg14 and Vps34, which facilitate the PI3P synthesis, strongly inhibits autophagosome fusion with the vacuole, suggesting PI3P serves as a prerequisite for the assembly of this tethering complex (Bas et al., 2018). Ypt7 subsequently binds to the two Ypt7-binding sites at the two ends of the HOPS complex to catalyze its flexible bending for driving the autophagosome-vacuole fusion (Figure 1D, left) (Balderhaar and Ungermann, 2013).

In mammalian cells, autophagosome-lysosome fusion is mediated by a SNARE complex consisting STX17-SNAP29-VAMP7/8 (Figure 1D, middle; Itakura et al., 2012). STX17 is recruited to autophagosomes via binding to the immunity-related GTPase M (IRGM) and LC3 on the autophagosomes (Kumar S. et al., 2018). Interestingly, STX17 interacts with all the HOPS subunits, and STX17 knockdown leads to improper autolysosome formation (Jiang et al., 2014). Another SNARE complex STX7-SNAP29-YKT6 also contributes to the fusion process, and the HOPS complex is likely to be recruited by STX7 (Jiang et al., 2014; Matsui et al., 2018). Different to yeast, it is reported that RAB7 and RAB2, which localize to the vacuole and autophagosomes, respectively, bind to the HOPS complex to trigger the autophagosome-lysosome fusion (Fujita et al., 2017; Figure 1D, middle).

Multiple SNAREs have been identified in *Arabidopsis*, but the involvement and underlying mechanisms of SNAREs in autophagy are largely unexplored (Fujiwara et al., 2014). In *Arabidopsis*, the VTI family (VTI11/12/13), the homologs of yeast Vti1, functions as Qb-SNARE and other associated Q-SNAREs have been identified via an interactomic approach (Surpin et al., 2003; Fujiwara et al., 2014). VTI11 interacts with syntaxin of plants 2 (SYP2), SYP5 and VAMP7 to form a Q-SNARE complex, while VTI12 assembles with SYP4, SYP6, and YKT6 (Figure 1D, right; Surpin et al., 2003; Fujiwara et al., 2014). These two Q-SNAREs participate in *trans-*Golgi networking and vacuole trafficking, as well as vacuole biogenesis (Ebine et al., 2008; Zouhar et al., 2009). The possible involvement of these SNARE proteins in plant autophagy is supported by the observation that both *vti11* and *vti12* mutants exhibit autophagy-associated phenotypes and abnormal autophagosomes are accumulated in *vti1* (Surpin et al., 2003). On the other hand, one Q-SNARE subunit, VAMP713 (vesicle-associated membrane protein 713), has been shown to interact with the HOPS complex to mediate vacuole fusion together with RAB7 (RabG3f) in plants (Takemoto et al., 2018). In addition, another Q-SNARE SYP22 binds the core subunit VPS33 of HOPS complex to mediate vacuolar fusion (Brillada et al., 2018). Importantly, proper localization of both VPS33 and the unique subunit VPS41 of HOPS complex to the vacuole is VTI11-dependent (Brillada et al., 2018). Therefore, it is very likely that a coordination between the HOPS complex and SNARE complex also operates in plant cells for heterotypic fusion with the vacuole membrane. Further studies are needed to investigate how they assemble and coordinate to tether the autophagosome and vacuole membrane (Figure 1D, right).

## Conclusion and Future Perspectives

The *de novo* formation of autophagosomes needs drastic lipid synthesis, transfer, exchange, and fusion to accomplish its fate into the vacuole for cargo sequestration and degradation. Sophisticated regulation mechanisms and machineries are utilized to shape the nascent membrane into a mature autophagosome timely and spatially, particularly with the coordination between the core ATG proteins and conserved membrane tethering complexes derived from the endomembrane system, thereby fulfilling the needs of different cell types under specific developmental processes or unfavorable conditions. A recent study also highlighted a role of the endocytic machinery in mediating autophagosome initiation at the ER-PM contact sites (Wang et al., 2019). Nevertheless, the diversity and conservation of regulatory mechanisms, models and novel tools learnt from yeast and mammalian systems, provides useful future insights for plant autophagy research. Autophagy has been implicated to function in unique plant cell types for plant development and growth. Therefore, future investigations in the autophagosome biogenesis in plant cells should provide valuable information for crop engineering to improve nutrient utilization and yield.

## Author Contributions

C-LW, YQ, and XZ designed the concept and the organization of the manuscript, and wrote and edited the manuscript.

## Conflict of Interest

The authors declare that the research was conducted in the absence of any commercial or financial relationships that could be construed as a potential conflict of interest.
